# Effects of Increasing the Orthodontic Forces over Cortical and Trabecular Bone during Periodontal Breakdown—A Finite Elements Analysis

**DOI:** 10.3390/medicina59111964

**Published:** 2023-11-07

**Authors:** Radu-Andrei Moga, Cristian Doru Olteanu, Mircea Daniel Botez, Stefan Marius Buru, Ada Gabriela Delean

**Affiliations:** 1 Department of Cariology, Endodontics and Oral Pathology, School of Dental Medicine, University of Medicine and Pharmacy Iuliu Hatieganu, Str. Motilor 33, 400001 Cluj-Napoca, Romania; ada.delean@umfcluj.ro; 2 Department of Orthodontics, School of Dental Medicine, University of Medicine and Pharmacy Iuliu Hatieganu, Str. Avram Iancu 31, 400083 Cluj-Napoca, Romania; 3 Department of Structural Mechanics, School of Civil Engineering, Technical University of Cluj-Napoca, Str. Memorandumului 28, 400114 Cluj-Napoca, Romania; mircea.botez@mecon.utcluj.ro (M.D.B.); marius.buru@mecon.utcluj.ro (S.M.B.)

**Keywords:** bone loss, failure criteria, periodontal breakdown, orthodontic force, Finite Elements Analysis, orthodontic movements

## Abstract

*Background and Objectives:* Herein we used numerical analysis to study different biomechanical behaviors of mandibular bone subjected to 0.6 N, 1.2 N, and 2.4 N orthodontic loads during 0–8 mm periodontal breakdown using the Tresca failure criterion. Additionally, correlations with earlier FEA reports found potential ischemic and resorptive risks. *Materials and Methods*: Eighty-one models (nine patients) and 243 simulations (intrusion, extrusion, rotation, tipping, and translation) were analyzed. *Results:* Intrusion and extrusion displayed after 4 mm bone loss showed extended stress display in the apical and middle third alveolar sockets, showing higher ischemic and resorptive risks for 0.6 N. Rotation, translation, and tipping displayed the highest stress amounts, and cervical-third stress with higher ischemic and resorptive risks after 4 mm loss for 0.6 N. *Conclusions:* Quantitatively, rotation, translation, and tipping are the most stressful movements. All three applied forces produced similar stress-display areas for all movements and bone levels. The stress doubled for 1.2 N and quadrupled for 2.4 N when compared with 0.6 N. The differences between the three loads consisted of the stress amounts displayed in color-coded areas, while their location and extension remained constant. Since the MHP was exceeded, a reduction in the applied force to under 0.6 N (after 4 mm of bone loss) is recommended for reducing ischemic and resorptive risks. The stress-display pattern correlated with horizontal periodontal-breakdown simulations.

## 1. Introduction

Bone structure (i.e., cortical, and trabecular bone) along with periodontal ligament (PDL) are the surrounding supporting tissues of the tooth and directly influenced by both orthodontic forces and the periodontal breakdown process [1,2,3]. The two components of bone should be analyzed together since clinically they behave as a continuum and their biomechanical behavior in both the intact and reduced periodontium is multifactorial, depending on the cortical and trabecular structural continuum, material and structural properties, and internal micro-architectural changes [3,4,5,6,7,8,9,10]. Trabecular bone is better vascularized and innervated (also holding the bone marrow, with a regenerative function) than the cortical component (with more structural support and a protective function) [3,7,8,11].

Bone is a ductile-resemblance material (with a brittle flow mode), showing a high ability to absorb and dissipate stresses and elastically deforming and recovering without fractures/destructions [2,8,10,12,13,14]. This behavior under applied loads produces temporary circulatory disturbances, that if maintained for a longer period of time can lead to ischemia and resorptive processes (through internal micro-architectural changes) [2,8,10,12,13,14,15,16]. The circulatory disturbances from the bone also affect PDL, dental pulp and the neuro-vascular bundle (NVB) [2,10,12,13,14,17,18] with a higher or lower amplitude depending on the anatomy and tissular integrity [9].

The circulatory disturbances trigger the orthodontic movements and remodeling of support tissues with limited effects in the intact periodontium (i.e., if light orthodontic forces are applied) [17,18,19], but variable and with unpredictable effects in the reduced periodontium (i.e., even under small loads) [17,18]. Despite numerous FEA (Finite Elements Method) studies regarding the effect of loads over the bone (bone–tooth [1,15,16,20,21,22] and bone–implant [3,7,9,23,24,25,26,27,28]) in the intact periodontium, no studies regarding the biomechanical behavior of bone subjected to a periodontal-breakdown process were found (except our team’s previous studies [2,10,12,13,14,17,18]).

The effects of orthodontic movements over the stress-extension areas in bone are variable (i.e., some are biomechanically more stressful), with various inconclusive reports of intrusion/extrusion [22], and translation and tipping [1,20,21] to be more stressful for the intact periodontium. No information was found for the reduced periodontium except our previous comparative studies reporting for both the intact and reduced periodontium that rotation and translation [2,10,12,13,14,17,18] are more stressful than the other movements, with increasing effects correlated with bone-loss progression.

Clinically during orthodontic treatment various levels of bone loss are found, signaling various levels of ischemic and resorptive risks that could alter the prognosis of the treatment if not managed with care. The only available noninvasive method of investigation for their biomechanical behavior is FEA analysis (i.e., a mathematically based algorithm method allowing an individual assessment of each component of a living structure) [2,10,12,13,14,17,18,23,24,27,29].

The FEA method is widely used in the engineering field with excellent accurate results, since its use is well documented and correlated with the type of analyzed material (i.e., each material has a specific designed failure criteria that better describes its biomechanical behavior, e.g., ductile, brittle, liquid, or gas according to yielding theory [10,12,13,14]). There are reports that only an FEA numerical simulation enables accurate biomechanical studies assessing and predicting stress distribution in living dental tissues [23,24,27,29].

In dental fields there are numerous FEA studies [1,3,7,9,15,16,20,21,22,23,24,25,26,27,28,29,30,31,32,33,34,35,36,37,38,39], but without any correlation with the type of analyzed material or with a maximum physiological hydrostatic pressure (MHP) of 16 KP (that if exceeded triggers ischemic and resorptive internal micro-architectural changes). These studies [1,3,7,9,15,16,20,21,22,23,24,25,26,27,28,29,30,31,32,33,34,35,36,37,38,39] reported various qualitative and quantitative contradicting results [2,10,12,13,14,17,18,19] from one study to another and with clinical known data. The previous studies [2,10,12,13,14,17,18] by our team were the first to address these issues proving that the FEA method could provide excellent accurate results in dental fields if some requirements are followed (i.e., proper failure criteria, boundary conditions, anatomically accurate 3D models, MHP correlation [3,23,24,27,29]). Thus, the Tresca failure criterion was reported to be better suited for the ductileness of dental tissues, where in the intact periodontium up to 1.2 N is safe to be applied, while in the reduced periodontium forces up to 0.6 N are better suited [2,10,12,13,14,17,18].

The well-vascularized dental tissues (PDL, NVB and dental pulp) are more sensitive to ischemia and resorptive process than the less-vascularized ones (dentine, cementum, cortical and trabecular bone), that are capable of withstanding a higher quantitative stress for a longer period. Thus, this is the explanation for the biomechanical behavior of the absorption–dissipation mechanism under light orthodontic forces that determine stresses lower than MHP in PDL, NVB and dental pulp, and higher quantitative values in dentine, cement, and bone [2,10,12,13,14,17,18].

Nevertheless, the comparative biomechanical behavior of the bone as a continuum during periodontal breakdown under the five orthodontic movements (intrusion, extrusion, tipping, rotation, and translation) and subjected to progressive increasing orthodontic loads has not been yet studied (despite its clinical significance). Moreover, to justify the accuracy of the results, the validation process needs to be performed through correlations with other numerical studies and clinical data (issues rarely touched upon in the research flow [1,3,7,9,15,16,20,21,22,23,24,25,26,27,28,29,30,31,32,33,34,35,36,37,38,39] but here addressed).

For enabling the correlations of the results, only four bone–tooth numerical studies [1,20,21,22] were found, for the intact periodontium and with one or two movements, reporting contradicting results: intrusion/extrusion vs. tipping as more stressful, variable amounts of stress and extension areas for the same movements and applied forces, PDL stresses exceeding the 16 KPa of MHP, no correlations with the MHP and no mention of the failure-criteria suitability issues.

Our step-by-step previous research [2,10,12,13,14,17,18] investigated the employment of proper failure-criteria material types based on investigating each component of dental tissues, as well as their biomechanical behavior during various levels of bone loss when subjected to orthodontic loads and movements. We [2,10,12,13,14,17,18] reported the Tresca criterion (designed for ductile non-homogenous materials) to be the suitable criterion for dental tissues. Another assessed issue was related to the maximum amount of orthodontic load that can be applied during orthodontic movements to each component of dental tissues in order to avoid ischemic and resorptive risks, since no studies investigating the gradual horizontal breakdown employing the Tresca criterion were found in the current research flow. The results reported that, in the intact periodontium, 0.6–1.2 N is relatively safely applied, while after 4 mm of bone loss forces of 0.2–0.6 N are recommended. The previous study [2] regarding mandibular bone reported Tresca as being the most suitable criterion for bone, by comparing five of the most-used failure criteria in various bone loss levels and under 0.5 N. However, no data regarding ischemic and resorptive risks related to the biomechanical behavior of applied loads in periodontal breakdown were found.

The goals of our study were (a) to assess the biomechanical behavior of cortical and trabecular bone as a continuum subjected to increasing orthodontic forces during horizontal periodontal breakdown; (b) to assess the differences between orthodontic movements and loads when subjected to increasing loads; (c) to correlate the results with other FEA-related reports of dental tissues for identifying potential resorptive and ischemic risks.

## 2. Materials and Methods

This FEA numerical analysis is part of a stepwise larger research project [2,10,12,13,14,17,18] (with the clinical protocol no.158, 2 April 2018) that investigated the biomechanical behavior of teeth and surrounding supporting tissues under light orthodontic forces in both intact and reduced periodontium.

Herein numerical analysis was performed over eighty-one models of the second mandibular premolar in 243 simulations, with a sample size of nine (nine models from nine patients). Regarding the sample size it must be emphasized that FEA numerical analyses [1,3,7,9,15,16,20,21,22,23,24,25,26,27,28,29,30,31,32,33,34,35,36,37,38,39] usually use a sample size of one (one model from one patient) since larger possibilities of changing the boundary conditions allow many simulations. Thus, the sample size of nine used here was found acceptable.

The inclusion criteria were intact arches, intact teeth, no malposition, intact or slightly reduced bone loss, non-inflamed periodontium, availability for regular checkups, indication of orthodontic treatments. The contraindications were in opposition to the above. In the research project more patients were initially included; however, at the end only nine qualified (four males/five females, mean age 29.81 ± 1.45).

Each patient received a CBCT examination (ProMax 3DS, Planmeca, Helsinki, Finland, voxel size 0.075 mm) of the mandibular region (with the two molars and premolars).

**Figure 1:**
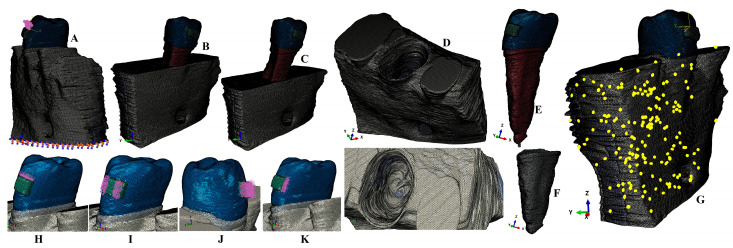
Mesh model: (**A**)—2nd lower right premolar model with intact periodontium and applied vectors (encastered model base and extrusion loads), (**B**)—4 mm bone loss, (**C**)—8 mm bone loss, (**D**)—intact bone structure (with cortical and trabecular components), (**E**)—2nd lower premolar with enamel, bracket, dentine, pulp, and NVB, (**F**)—intact PDL, (**G**)—element warnings of the cortical and trabecular components (yellow spots), applied vectors: (**H**)—intrusion, (**I**)—rotation, (**J**)—tipping, (**K**)—translation.

The grey shades DICOM images were manually segmented using Amira 5.4.0 (Visage Imaging Inc., Andover, MA, USA), allowing the identification of small tissues that were not recognized by the automated algorithm. Thus, our models contained: enamel; dentin-cementum; dental pulp; NVB; PDL; cortical bone; and trabecular bone. The base of a stainless-steel bracket was manually reconstructed on the vestibular surface of the second premolar. The models guarded only the second lower premolar while the alveolar socket of the other teeth was filled with bone (Figure 1). These nine models had various but limited levels of bone loss (i.e., in the cervical third of the alveolar socket). The bone loss and missing PDL were manually reconstructed, thus obtaining nine models with intact periodontium.

Each of these 9 models was then subjected to a gradual horizontal reduction of bone and PDL of around 1 mm, obtaining a periodontal breakdown of 1–8 mm (for each patient 9 models were obtained with various bone losses, totaling 81 3D models). All these models were anatomically correct (e.g., 5.06–6.05 million C3D4 tetrahedral elements, 0.97–1.07 million nodes, and a global element size of 0.08–0.116 mm, for the 9 intact periodontium models). The PDL had a variable thickness of 0.15–0.225 mm, including NVB, while cementum was reconstructed as dentine (since they have similar physical properties, and their separation was found to be impossible).

The mesh models displayed various surface irregularities (201 element warnings meaning 0.0039% from a total of 5,117,355 elements of the bone continuum) and no error warnings (Figure 1G, yellow spots). All these surface irregularities were in non-essential areas not interfering with the quasi-continuity of the stress-affected areas. Moreover, each software had internal checking and testing algorithms that did not allow a new step if the earlier one had not been passed.

The numerical simulations were performed using Abaqus 6.13-1 (Dassault Systèmes Simulia Corp., Maastricht, The Netherlands), employing the Tresca (maximum shear stress) failure criterion suited for ductile materials with physical properties [2,10,12,13,14,17,18] (Table 1) similar to other FEA numerical studies. The simulated orthodontic movements were intrusion, extrusion, rotation, tipping, and translation under three applied forces (0.6 N approx. 60 g, 1.2 N approx. 120 g, and 2.4 N approx. 240 g) at the bracket base (Figure 1). The base of the model had zero displacements (encastered).

The three forces were selected based on the fact that they are considered relatively safe to apply in both intact and reduced periodontium and for our being able to corelate these simulations with previous ones investigating other tissular components (PDL, dental pulp, NVB, bone) [2,10,12,13,14,17,18].

The boundary conditions included homogeneity, linear elasticity, and isotropy similar with the other FEA numerical analyses mentioned above.

The results were color-coded projections of various colors and extension areas of the shear stress (qualitative and quantitative), that were compared and correlated with both previous studies of our team [2,10,12,13,14,17,18] and with the four identified bone–tooth research papers [1,20,21,22].

## 3. Results

The analysis herein was performed over eighty-one models in a total of 243 FEA simulations, with no influence whatsoever due to gender, age, or periodontal status.

There was a constant and coherent visible pattern of stress increase and extension areas for all movements and loads strictly correlated with the progression of the periodontal breakdown. Rotation seemed to be the most stressful among the five orthodontic finemovements for the mandibular bone.

**Figure 2:**
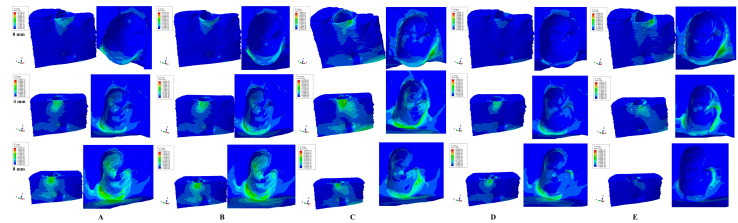
Comparative stress display of the five movements under 0.6 N in intact, 4 mm, and 8 mm periodontal breakdown, vestibular and interior alveolar socket view: (**A**)—extrusion, (**B**)—intrusion, (**C**)—rotation, (**D**)—tipping, (**E**)—translation.

**Figure 3:**
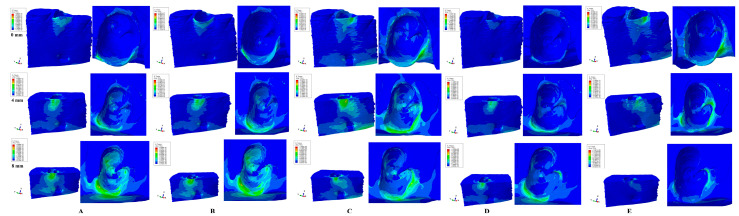
Comparative stress display of the five movements under 1.2 N in intact, 4 mm, and 8 mm periodontal breakdown, vestibular and interior alveolar socket view: (**A**)—extrusion, (**B**)—intrusion, (**C**)—rotation, (**D**)—tipping, (**E**)—translation.

Qualitatively, FEA simulations displayed similar color-coded projections of stress distribution areas (i.e., Figure 2 and Figure 3) for all three forces independently of their amount (i.e., 0.6 N, 1.2 N and 2.4 N).

Quantitatively, the amount of displayed stress doubled (for 1.2 N) and quadrupled (for 2.4 N) when compared with 0.6 N of force (Table 2). The quantitative increase rate (i.e., a%, m%, c%) was similar for all three forces independently of the levels of bone loss displaying a gradual increase up to a doubling of the amount of stress (at 8 mm of loss) when compared with the intact periodontium.

Both qualitatively and quantitatively, vestibular cervical stress was the highest for all three movements (rotation, translation, and tipping) and forces, seeming to display higher risks of ischemia and further resorptive processes. The extension of vestibular cervical-stress areas progressively increased with bone loss.

As expected, all amounts of stress exceeded the 16 KPa of maximum physiological hydrostatic pressure (MHP) (e.g., 2.34–25.15 times for 0.6 N) (Table 2) since the mandibular bone is anatomically less vascularized than other tissues (i.e., PDL, dental pulp, the neuro-vascular bundle), and its ischemic risks are smaller when compared with these above tissues. Nevertheless, these amounts of stress (Table 2) were lower than acknowledged bone physical properties: cortical bone—16.7 GPa of compressive modulus and 157 MPa of compressive strength; trabecular/cancellous bone 0.155 GPa of compressive modulus and 6 MPa of compressive strength [3,7,9,23,24,25,26,27,28].

Rotation, translation, and tipping movements produced the highest amounts of stress both inside and outside the alveolar socket (after 4 mm of bone loss) seeming to be prone to increased ischemic and resorptive risks when compared with the intrusion and extrusion movements. Outside the alveolar socket, a progressive extension of vestibular stress to the middle- and apical vestibular third was seen for all five movements correlated with bone loss. Inside the alveolar socket, in the intact periodontium, all five movements produced limited vestibular cervical stress with no visible (except rotational) stresses in the apical and middle third. However, with the progression of periodontal breakdown (especially after 4 mm of loss), stress areas were extended to the entire inside surface of the alveolar socket. Thus, this confirms a correlation between bone loss and increased risks of apical and middle third ischemic and resorptive processes for the alveolar socket after 4 mm of loss even for 0.6 N of applied force.

In both the intact and reduced periodontium, the 3D models displayed stress areas found at the base, since it was encastered and with zero displacements (i.e., simulating mandibular stiffness). These areas were more visible after 4 mm of bone loss due to the reduction in the bone height and periodontal ligament (i.e., surrounding supporting tissues) with no influence, however, over the stress distribution in the alveolar socket. These areas of stress near the bases of the models were expected (similar to other numerical analyses).

Extrusion (Figure 2A and Figure 3A) displayed limited vestibular cervical stress (where the bone is thinner) for the intact periodontium, with a progressive extension to the middle and apical third of the alveolar socket from 4 to 8 mm of periodontal breakdown. A bone loss level of 8 mm displayed the highest extension of stress areas to the entire alveolar socket. Quantitatively, for 0.6 N of force the apical third stress in the alveolar socket exceeded the MHP by four times. The cervical third stress had a limited increase interval of 149.83–168.34 KPa (for 0.6 N, 0–8 mm bone loss), almost ten times higher than the MHP, signaling potential ischemic and resorptive risks. On the lingual side of the model, the base area displayed limited stress areas (due to boundary conditions with zero displacement) correlated with bone loss, with no significance whatsoever for the alveolar-socket stress display.

Intrusion (Figure 2B and Figure 3B) displayed a similar behavior to the extrusion movement.

Rotation (Figure 2C and Figure 3C) displayed mostly vestibular cervical stress for the intact periodontium, with a progressive but limited extension (much smaller than intrusion/extrusion) to the apical third after 4 mm of loss. It also displayed the highest and most extended stress areas (both vestibular and lingual) at the base of the models due to the assumption of zero displacements (with no influence over the stress distribution in the alveolar socket). Quantitatively, rotation produced the highest amount of stress among the five movements.

Tipping (Figure 2D and Figure 3D) movement showed only vestibular cervical stress in the intact periodontium. However, along with progression of bone loss there was a visible extension of stress to the vestibular and lingual middle and apical third of the socket. On the lingual and vestibular sides near to the base of the model, limited visible stresses were displayed due to assumed boundary conditions.

Translation (Figure 2E and Figure 3E) movement displayed limited cervical-third vestibular stress in the intact periodontium, with a progressive extension to the proximal and lingual cervical-third sides of the socket correlated with bone loss.

The quantitative amounts of stress provided by Tresca failure criterion simulations (specific for non-homogenous ductile resemblance materials with a certain brittle flow mode [2,10,12,13,14]) fell within the range of 15–30% reported in the literature (i.e., ours being around 15%) [2].

## 4. Discussion

These 243 FEA simulations performed over eighty-one models aimed to assess the biomechanical behavior of cortical and trabecular bone as a continuum subjected to increasing orthodontic forces during horizontal periodontal breakdown. Additionally, the aim was to assess the differences between orthodontic movements when subjected to different loads, as well as to correlate results with other FEA-related reports of dental tissues for finding potential resorptive and ischemic risks. It must be emphasized that herein being the first investigation into these issues, and since the current research flow is scarce, the only available sources of comparison were our previous research [2,10,12,13,14,17,18] and the four bone–tooth studies [1,20,21,22].

This manuscript further develops the previous published research [2] (being a step-by-step developed study). The previous research focused on selecting suitable failure criteria for the numerical study of bone (as a structural continuum) by employing five failure criteria used in dental FEA analysis, 0.5 N applied force, five movements and 0–8 mm periodontal breakdown. The qualitative and quantitative results provided by the simulations prove that only failure criteria for ductile materials are suitable (i.e., giving accurate results when compared with the other three) for bone study, with in particular Tresca being the best option. We must emphasize that only our studies employed Tresca, we being the first to use it in dental field research.

On the other hand, our research took the next logical step in the study of bone, investigating how much the increase in orthodontic force affects both qualitative and quantitative results. Thus, we employed the previous proven Tresca (best suitable criterion) and, under 0.6 N, 1.2 N, and 2.4 N, we analyzed how the changes affect the intact bone and the reduced periodontium, and what the ischemic and resorptive risks are.

In our simulations, the color-coded projections of the stress-distribution areas (qualitative results) in both the intact and reduced periodontium were similar for all three applied forces. However, the amounts of stress displayed by these areas increased along with the applied force (doubled for 1.2 N and quadrupled for 2.4 N when compared with 0.6 N). These above results suggest that the differences between these light orthodontic forces consist of stresses (quantitative) displayed in the color-coded stress areas, while their location and extension remain constant (qualitative). There seems to be a correlation between the progression of bone loss (i.e., surrounding supporting tissues), extension of stress areas to the alveolar socket, and increase in displayed amounts of stress (in agreement with previous data [2,10,12,13,14,17,18]).

Previous studies investigating PDL, dental pulp and NVB behavior during 0–8 mm bone loss under the same five orthodontic movements and three forces (correlated with the MHP) reported 0.6–1.2 N to be safe for the intact periodontium and 0.2–0.6 N safe for various levels of bone loss [17,18]. The results herein agree with these observations.

The biomechanically similar stress display areas in the intrusion and extrusion movements during gradual horizontal periodontal breakdown are due to the periodontal ligament functions (absorption–dissipation stress ability) [2,10,14,17,18]. As the surrounding supporting tissues (bone and ligament) are reduced, the stress distribution changes so that the entire tooth alveolar socket absorbs these stresses [2,10,14,17,18]. We must emphasize that only intrusion and extrusion, among the five movements, displayed such a rapid extension of the stress areas in the apical and middle third of the alveolar socket after 4 mm of bone loss, in agreement with previous studies [2,10,14,17,18]. This behavior seems to lead to the conclusion that with bone loss the risks of apical and middle-third alveolar-socket ischemia and resorptive processes for these two movements increase when compared with the other three movements (due to better vascularization of this area—NVB, dental pulp, perforating vessels) [2,12,13,17,18]. Moreover, quantitatively, in the apical third of the alveolar socket the amount of stress displayed for 0.6 N has a range of 61.33–67.3 KPa for 4–8 mm of loss, around four times higher than the physiological MHP of 16 KPa. Nevertheless, the cervical-third stress is nine times higher (i.e., 0.6 N and the intact periodontium) than MHP stress, but much lower when compared with the other three movements. This observation seems also to be supported by the clinical and anatomical data that the apical third of the alveolar socket has better vascularization than the cervical third [2,10,12,13,14,17,18].

Rotation, translation, and tipping displayed mainly vestibular cervical-third stress in the alveolar socket during the entire periodontal-breakdown simulation (due to PDL absorption–dissipation ability), leading to the conclusion that ischemic and resorptive risks are higher in these areas, in agreement with our previous studies [2,10,12,13,14,17,18]. Moreover, the amount of cervical stress for a rotational applied force of 0.6 N reached a maximum of 402.71 KPa for 8 mm bone loss (i.e., twenty-five times higher than the MHP). These findings agree with our earlier results [2]. On the other hand, from the clinical data, 0.6 N of force is considered to be light and safe to be used in the intact periodontium [19], but there are no available studies for the reduced periodontium, except our previous ones [2,10,12,13,14,17,18]. Thus, despite 0.6 N being considered safe for the intact periodontium, for the assessment of safety in the reduced periodontium we can only rely on data present in the current research flow (i.e., FEA studies) [2,10,12,13,14,17,18].

The above findings correlate with previous reports [2,12,13,15,16,17,18] of 0.6 N effects over PDL (higher risks of ischemia and further loss) and their recommendations [15,16,17,18] of a reduction to 0.2–0.4 N in the applied force in cases of periodontal breakdown (4–8 mm of loss). It also confirms a correlation between bone loss and increased risks of apical and middle-third ischemic and resorptive processes for the alveolar socket after 4 mm of loss even for 0.6 N of applied force. However, it must be emphasized that bone as a continuum is less vascularized than PDL and dental pulp–NVB and thus less prone to circulatory disturbances. Therefore, the maximum force safely applied in these tissues (i.e., PDL, pulp, tooth, bone, NVB) should be the one that is safe for the weakest component (i.e., PDL and NVB), and thus, previous recommendations [17,18] should be considered.

Another aspect that should be addressed is related to the fact that in both the intact and reduced periodontium, the models displayed stress areas found at the base, since it was encastered and with zero displacements (i.e., simulating mandibular stiffness). These areas were more visible after 4 mm of bone loss due to the reduction in bone height and periodontal ligament (i.e., surrounding supporting tissues) with no influence however over the stress distribution in the alveolar socket.

FEA analysis is the only possible method of individually investigating small and complex structures; thus, for correct results, all known issues related to the investigating method must be addressed. The selected failure criteria must be professionally designed for the analyzed material. The FEA studies [1,3,7,9,15,16,20,21,22,23,24,25,26,27,28,29,30,31,32,33,34,35,36,37,38,39] usually employed multiple failure criteria (Von Mises, maximum and minimum principal stress, hydrostatic pressure) without discussing their suitability for the analysis of dental tissues. This aspect was addressed only in our previous studies [2,10,12,13,14,17,18], reporting that dental tissues (PDL, dental pulp, NVB, dentine, cement, trabecular and cortical bone) possess ductile resemblance (with a certain brittle flow mode) with the Tresca (along with Von Mises) failure criterion being better suited. In previous research [2] bone was reported to display a ductile-resemblance biomechanical behavior (i.e., various recoverable elastic deformations when subjected to stresses, that totally returned to their original form after the forces disappeared) [3].

For validating FEA results, correlations with the 16 KPa of physiological maximum hydrostatic pressure values (i.e., about 80% of the systolic pressure) found in dental tissues must be performed. If the MHP is exceeded, circulatory disturbances, ischemia and resorptive processes are started. Thus, this shows the importance of considering the anatomical vascularization and innervation of these tissues. Most FEA studies [1,3,7,9,15,16,20,21,22,23,24,25,26,27,28,29,30,31,32,33,34,35,36,37,38,39] did not address this issue.

The stress distribution in the implant–bone interface area was widely investigated using FEA analysis (i.e., the only available method of study) [3,7,9,23,24,25,26,27,28]. These studies [3,7,9,23,24,25,26,27,28] used uniaxial loading, a sample size of one, Von Mises criteria (closely related with Tresca), and reported bone cervical-third stress concentrations, which is in line with the findings herein for intrusion. However, due to important biomechanical differences (mainly lack of PDL and higher applied forces: 3–10 N [7,28]; 40–800 N [3,9,23,24,25,26,27]) significantly changing the results, the quantitative values cannot be compared despite the similarity in boundary conditions and failure criteria.

The failure criterion employed in these FEA studies [3,7,9,20,21,22,23,24,25,26,27,28] was Von Mises (overall stress, for ductile homogenous materials), which is qualitatively similar with Tresca (shear stress, for non-homogenous ductile material but with a certain brittle flow mode), but quantitatively 15–30% lower. The Von Mises criterion is suited for bone–implant and bone–tooth FEA studies [2,10,12,13,14,17,18]. However, Tresca is better suited for bone–tooth models since these are considered non-homogenous ductile materials with a brittle flow mode [2,10,12,13,14,17,18].

No FEA bone–tooth studies employing the Tresca failure criterion were found. However, four FEA intact periodontium tooth–bone studies using the Von Mises criterion and similar boundary conditions were found [1,20,21,22].

Merdji et al. [20] (lower third molar, intact periodontium, single model, sample size of one, Von Mises criterion, intrusion: 10 N, tipping/translation: 3 N, bone: 142,305 elements, global element size: 0.25–1 mm), qualitatively reported (similar with the results herein) cervical-third stress for all three movements. Qualitatively, Merdji et al. [20] reported that the stress display was both on the vestibular and lingual cervical third of the alveolar socket since both sides of the bone were of equal thickness (probably due to three-rooted anatomy and artificial anatomical positioning), and from this point of view is closer to bone–implant [3,7,9,23,24,25,26,27,28] stress display. In our models, the correct anatomical reconstruction avoided these shortcomings (the second premolar displayed a vestibular alveolar socket wall thinner than the lingual one); thus, the stress distribution areas were found toward the thinner and less resistant wall.

Quantitatively, Merdji et al. [20] reported 10.5 Mpa for 10 N of intrusion, 11.5 Mpa for 3 N of tipping, and 16.83 Mpa for 3 N of translation, while in our study 0.6 N produced 149.83 Kpa/0.149 Mpa (intrusion), 146.53 Kpa/0.146 Mpa (tipping), and 185.28 Kpa/0.185 Mpa (translation), and 2.4 N displayed 599.32 Kpa/0.599 Mpa (intrusion), 586.12 Kpa/0.586 Mpa (tipping), and 741.14 Kpa/0.741 MPa (translation).

We assumed that in the appearance of these differences an important role was played both by boundary conditions (global element size 0.25–1 mm and 142,305 elements [20] vs. global element size 0.08–0.116 mm and 5,117,355 elements herein) and the anatomy of the model (idealized third molar [20] vs. our anatomically accurate second premolar).

Field et al. [21] (intact periodontium, two models, sample size of two, Von Mises criterion, tipping: 0.5 N, canine model: 23,565 elements, incisor–canine–first premolar model: 32,812 elements, global element size: 1.2 mm) qualitatively reported comparable results (such as extension and topography) to this study, but with different color-coded codification meaning high stress intensity (contradicting the color codification herein). We must emphasize that Field et al. [21] applied 0.35 N/0.5 N of tipping, a force considered light [19], reporting extended red high stresses [21] vs. limited-extension blue-green lower stresses in our simulations (much closer to the clinical behavior of a reduced force).

Field et al. [21] reported quantitative results for bone cervical-third stress of 236.3–287.8 KPa [21], vs. 146.53 KPa in our simulations.

We assumed that these differences were due to modeling issues and boundary conditions (i.e., global element size 1.2 mm and 23,565–32,812 elements [21] vs. global element size 0.08–0.116 mm, 5.06–6.05 million elements, and 0.97–1.07 million nodes in our simulations). In support of these assumptions, we consider relevant the reported [21] amount of 32 KPa of hydrostatic pressure in the apical third of PDL and Von Mises stresses of 235.5–324.5 KPa in the entire PDL, exceeding by far the reported MHP of 16 KPa, suggesting high ischemia and resorptive risks for a light force of 0.35 N/0.5 N, contradicting both clinical data [19] and other FEA studies (i.e., 0.5 N proven to be safely applied in both PDL and pulp–NVB up to 8 mm of loss with amounts of stress lower than MHP) [17,18]. Field et al. [21] did not perform correlations with physiological MHP and reported significant qualitative and quantitative differences between the biomechanical behavior of the two models (i.e., higher extension of the stress areas for the multi-teeth model when compared with the single-tooth model). In our simulations both qualitative and quantitative results were lower than those Field et al. [21] reported, and we expect that the same pattern should be displayed by a multi-teeth model.

Shaw et al. [22] (upper incisor, intact periodontium, one model, sample size of one, Von Mises criterion, intrusion, extrusion, tipping, translation and rotations, model: 11,924 elements and 20,852 nodes) reported lower amounts of cervical stress (i.e., intrusion/extrusion 1.664 KPa, translation 0.6 KPa, tipping 0.54 KPa and rotation 0.015 KPa), intrusion and extrusion as the most stressful movements, and comparable stress-display areas. We assumed that these differences were due to modeling issues and boundary conditions.

Shetty et al. [1] (upper 1st molar, intact periodontium, one model, sample size of one, Von Mises criterion, intrusion and tipping: 150 N, model: 30,838 nodes and 167,089 elements) quantitatively reported 1.33–1.95 MPa for intrusion and 2.16–8.15 MPa for tipping, with tipping as more stressful than intrusion (in agreement with the results herein) and qualitatively displaying extended stress areas in the entire alveolar socket for both movements (and contradicting our results herein). It must be emphasized that these models [1] are not anatomically accurate and were simplified (i.e., lower numbers of nodes and elements) along with other boundary conditions; thus, the differences (i.e., high amounts of stress and extension areas) could be explained. In the simulations here, there were no qualitative differences between 0.6 and 2.4 N of force, so we expect that this pattern should be kept for higher forces. Nonetheless, the quantitative results here rapidly increased so higher amounts of stress could be possible under higher forces. However, the fundamental issues reside in the appliance time and the integrity of supporting tissues (more than 0.6 N is prone to ischemic and resorptive risks in the reduced periodontium if kept for longer periods of time).

The limits of FEA studies are related to the anatomical accuracy of the model and boundary conditions, as well as to the selection of the proper failure criteria. It must be emphasized that another limit is that FEA studies cannot entirely reproduce clinical situations and that clinically pure movements rarely happen (mostly, a combination of them is met).

The proper failure criteria are related to the type of analyzed material (ductile or brittle, homogenous–nonhomogeneous), since each criterion was specially designed for a certain type of biomechanical behavior. The Tresca criterion is the single criterion suited for all components of teeth and the surrounding supporting tissues (proven by previous FEA studies) [2,10,12,13,14,17,18].

The boundary conditions used by FEA studies [1,3,7,9,15,16,20,21,22,23,24,25,26,27,28,29,30,31,32,33,34,35,36,37,38,39] are generally identical: isotropy, elasticity, and homogeneity, despite the anatomical tissues being anisotropic, non-homogenous and nonlinear elastic. However, it must be emphasized that from a biomechanical point of view, under small, applied forces (around 1 N) all materials/tissues display linear elasticity [2,10,12,13,14,17,18]. Thus, based on the above, the use of light orthodontic forces is prone to produce more correct results in FEA simulations than higher forces. Regarding the homogeneity issues, by using a failure criterion such as Tresca specially designed for ductile non-homogenous materials, more accurate results are obtained when compared with other criteria such as Von Mises (ductile homogenous materials), maximum and minimum principal stresses (brittle) or hydrostatic pressure (liquids) [2,10,12,13,14,17,18].

Few FEA studies assessed the non-linear vs. linear elasticity issues, reporting quantitative differences for light orthodontic forces (1 N). Thus, Hemanth et al. [15,16] reported 20–50% less force needed for non-linear elasticity when compared to linear for obtaining the same results. Nevertheless, Hemanth et al. [15,16] employed a brittle material criterion (maximum and minimum principal stresses) for biomechanically analyzing PDL (which is ductile) under extremely small movements (intrusion and tipping), using a model with an idealized anatomy, all with a high potential of altering the accuracy of the results. More studies are needed for assessing this issue.

The anatomical accuracy of the analyzed model also has a potential for altering the accuracy of results (as previously shown). An FEA model should be based entirely on CBCT data, with a reduced voxel size (not artificially reconstructed based on a simplified idealized anatomy), and with a mesh displaying a large number of elements and nodes (e.g., the 5.06–6.05 million elements and 0.97–1.07 million nodes used herein vs. 142,305 elements [20]; 23,565–32,812 elements [21]; 30,838 nodes and 167,089 elements [1]; 148,097 elements and 239,666 nodes [15,16]; 11,924 elements and 20,852 nodes [22]); and a small global element size (e.g., 1.2 mm [21]; 0.25–1 mm [20] vs. the 0.08–0.116 mm used herein).

Anatomical correct 3D models are difficult to create (mostly based on a manual segmentation process for finding all tissular components); thus, most FEA studies use simplified and idealized anatomical models. Moreover, even these models are difficult to create. Thus, most of the FEA studies use only one model, from one patient (i.e., a sample size of one) [1,3,7,9,15,16,20,21,22,23,24,25,26,27,28,29,30,31,32,33,34,35,36,37,38,39]. Nevertheless, FEA analysis allows a great number of simulations and changing of boundary conditions which overcomes the inconvenience of having a small number of cases [1,3,7,9,15,16,20,21,22,23,24,25,26,27,28,29,30,31,32,33,34,35,36,37,38,39]. Based on the above, we found it to be acceptable to use nine patients (thus, a sample size of nine), having a total of eighty-one models and 243 simulations. The mesh models, especially those manually segmented, tend to display surface anomalies and irregularities (as with the models here), that usually do not interfere with the result accuracy since FEA software (Abaqus 6.13-1) has internal testing algorithms. We must emphasize that our models display such small, limited surface irregularities (Figure 1G, yellow spots) found in non-essential areas.

FEA studies (i.e., numerical simulations) are the only available method to individually study such small and complex structures as dental living tissues. However, despite the individual study of each component, clinically, all these components work and function as a continuum; thus, this shows the importance of correlations with MHP and between available studies. Both our results herein and our previous [2,10,12,13,14,17,18] studies were focused on addressing these issues for matching the numerical results with clinical data (since, due to misunderstanding of the FEA methodology [1,3,7,9,15,16,20,21,22,23,24,25,26,27,28,29,30,31,32,33,34,35,36,37,38,39], most of the dental FEA studies are not as accurate as those from the engineering field). Our research is the first in the above-mentioned direction; thus, we tried to explain the main issues regarding the use of FEA in dental studies for obtaining more accurate results and identifying and using one general single failure criterion (found to be Tresca [2,10,12,13,14,17,18]) for the study of all dental structures.

## 5. Conclusions

1. Both intrusion and extrusion after 4 mm of bone loss displayed extended stress display in the apical and middle third of the alveolar socket, seeming to show higher ischemic and resorptive risks for these areas even for 0.6 N.
2. Rotation, translation, and tipping displayed the highest amounts of stress and cervical-third stress with higher ischemic and resorptive risks after 4 mm of loss for 0.6 N.
3. Based on quantitative results, rotation, translation, and tipping seem to be more stressful when compared with intrusion and extrusion.
4. All three applied forces produced similar stress display areas for all movements and bone levels.
5. The amount of stress doubled for 1.2 N and quadrupled for 2.4 N when compared with 0.6 N.
6. The differences between the three orthodontic forces consist of stress (quantitative) displayed in the color-coded stress areas, while their location and extension (qualitative) remained constant in both the intact and reduced periodontium.
7. Since the MHP was exceeded in all simulations, a reduction in the applied force to under 0.6 N (after 4 mm of bone loss) is recommended for reducing the ischemic and resorptive risks.
8. The stress display pattern correlated with horizontal periodontal-breakdown simulations.

## 6. Practical Implications

Little information is available about the biomechanical behavior of bone as a continuum during periodontal breakdown. Moreover, the effects (distribution areas and amounts of stress) of various applied orthodontic forces are unknown. Thus, this research studied the effects of three orthodontic forces (0.6 N, 1.2 N and 2.4 N) over 0–8 mm bone loss during five orthodontic movements. It also correlated the results with other studies with the same boundary conditions but investigating dental pulp, the periodontal ligament, the neuro-vascular bundle and the surrounding bone, thus supplying a clearer and more comprehensive image of the entire biomechanical behavior of these tissues. The results are extremely important for both clinical practitioners and researchers. The stress distribution under intrusion and extrusion seems to favorize the ischemic and resorptive risks in the apical and middle third of the alveolar socket while rotation, translation, and tipping have a similar effect localized to the cervical third of the alveolar socket. Moreover, after 4 mm of bone loss a reduction in the amount of force to under 0.6 N is recommended. Both researchers and clinicians can benefit from the comprehensive image created by the FEA studies and better understand the advantages but also the limits of this method of investigation of small anatomical tissues.

## Figures and Tables

**Table 1:** Elastic properties of materials.

Material	Young’s Modulus, E (GPa)	Poisson Ratio, ʋ	Refs.
Enamel	80	0.33	[2,10,12,13,14,17,18]
Dentin/Cementum	18.6	0.31	[2,10,12,13,14,17,18]
Pulp	0.0021	0.45	[2,10,12,13,14,17,18]
PDL	0.0667	0.49	[2,10,12,13,14,17,18]
Cortical bone	14.5	0.323	[2,10,12,13,14,17,18]
Trabecular bone	1.37	0.3	[2,10,12,13,14,17,18]
Bracket (Stainless Steel)	190	0.265	[2,10,12,13,14,17,18]

**Table 2:** Maximum stress average values (KPa) produced by 0.6–2.4 N of orthodontic forces.

Resorption (mm)			0	1	2	3	4	5	6	7	8
Extrusion	0.6 N	a	37.46	43.43	49.40	55.36	61.33	62.82	64.31	65.80	67.30
		a%	1.00	1.16	1.32	1.48	1.64	1.68	1.72	1.76	1.80
		m	37.46	38.32	39.17	40.02	40.89	43.27	45.67	48.00	50.47
		m%	1.00	1.02	1.05	1.07	1.09	1.16	1.22	1.28	1.35
		c	149.83	153.25	156.67	160.09	163.51	164.71	165.92	167.12	168.34
		c%	1.00	1.02	1.05	1.07	1.09	1.10	1.11	1.12	1.12
	1.2 N	a	74.92	86.86	98.80	110.71	122.65	125.64	128.62	131.59	134.60
		a%	1.00	1.16	1.32	1.48	1.64	1.68	1.72	1.76	1.80
		m	74.92	76.63	78.34	80.04	81.77	86.54	91.34	96.00	100.95
		m%	1.00	1.02	1.05	1.07	1.09	1.16	1.22	1.28	1.35
		c	299.66	306.50	313.34	320.18	327.02	329.42	331.85	334.25	336.68
		c%	1.00	1.02	1.05	1.07	1.09	1.10	1.11	1.12	1.12
	2.4 N	a	149.84	173.71	197.59	221.42	245.30	251.28	257.23	263.18	269.19
		a%	1.00	1.16	1.32	1.48	1.64	1.68	1.72	1.76	1.80
		m	149.84	153.26	156.67	160.08	163.54	173.09	182.69	192.00	201.90
		m%	1.00	1.02	1.05	1.07	1.09	1.16	1.22	1.28	1.35
		c	599.32	613.01	626.69	640.37	654.04	658.85	663.70	668.50	673.36
		c%	1.00	1.02	1.05	1.07	1.09	1.10	1.11	1.12	1.12
Intrusion	0.6 N	a	37.46	43.43	49.40	55.36	61.33	62.82	64.31	65.80	67.30
		a%	1.00	1.16	1.32	1.48	1.64	1.68	1.72	1.76	1.80
		m	37.46	38.32	39.17	40.02	40.89	43.27	45.67	48.00	50.47
		m%	1.00	1.02	1.05	1.07	1.09	1.16	1.22	1.28	1.35
		c	149.83	153.25	156.67	160.09	163.51	164.71	165.92	167.12	168.34
		c%	1.00	1.02	1.05	1.07	1.09	1.10	1.11	1.12	1.12
	1.2 N	a	74.92	86.86	98.80	110.71	122.65	125.64	128.62	131.59	134.60
		a%	1.00	1.16	1.32	1.48	1.64	1.68	1.72	1.76	1.80
		m	74.92	76.63	78.34	80.04	81.77	86.54	91.34	96.00	100.95
		m%	1.00	1.02	1.05	1.07	1.09	1.16	1.22	1.28	1.35
		c	299.66	306.50	313.34	320.18	327.02	329.42	331.85	334.25	336.68
		c%	1.00	1.02	1.05	1.07	1.09	1.10	1.11	1.12	1.12
	2.4 N	a	149.84	173.71	197.59	221.42	245.30	251.28	257.23	263.18	269.19
		a%	1.00	1.16	1.32	1.48	1.64	1.68	1.72	1.76	1.80
		m	149.84	153.26	156.67	160.08	163.54	173.09	182.69	192.00	201.90
		m%	1.00	1.02	1.05	1.07	1.09	1.16	1.22	1.28	1.35
		c	599.32	613.01	626.69	640.37	654.04	658.85	663.70	668.50	673.36
		c%	1.00	1.02	1.05	1.07	1.09	1.10	1.11	1.12	1.12
Rotation	0.6 N	a	76.74	84.65	92.57	100.48	108.39	119.02	129.64	140.26	150.88
		a%	1.00	1.10	1.21	1.31	1.41	1.55	1.69	1.83	1.97
		m	76.74	84.65	92.57	100.48	108.39	119.02	129.64	140.26	150.88
		m%	1.00	1.10	1.21	1.31	1.41	1.55	1.69	1.83	1.97
		c	306.88	322.78	338.66	354.55	370.45	378.43	386.42	394.42	402.41
		c%	1.00	1.05	1.10	1.16	1.21	1.23	1.26	1.29	1.31
	1.2 N	a	153.49	169.30	185.14	200.95	216.78	238.03	259.27	280.51	301.77
		a%	1.00	1.10	1.21	1.31	1.41	1.55	1.69	1.83	1.97
		m	153.49	169.30	185.14	200.95	216.78	238.03	259.27	280.51	301.77
		m%	1.00	1.10	1.21	1.31	1.41	1.55	1.69	1.83	1.97
		c	613.76	645.55	677.33	709.10	740.90	756.86	772.85	788.83	804.82
		c%	1.00	1.05	1.10	1.16	1.21	1.23	1.26	1.29	1.31
	2.4 N	a	306.98	338.59	370.27	401.90	433.57	476.06	518.54	561.02	603.54
		a%	1.00	1.10	1.21	1.31	1.41	1.55	1.69	1.83	1.97
		m	306.98	338.59	370.27	401.90	433.57	476.06	518.54	561.02	603.54
		m%	1.00	1.10	1.21	1.31	1.41	1.55	1.69	1.83	1.97
		c	1227.53	1291.10	1354.66	1418.21	1481.80	1513.73	1545.70	1577.66	1609.63
		c%	1.00	1.05	1.10	1.16	1.21	1.23	1.26	1.29	1.31
Tipping	0.6 N	a	73.27	78.48	83.70	88.92	94.14	97.81	101.50	105.17	108.85
		a%	1.00	1.07	1.14	1.21	1.28	1.33	1.39	1.44	1.49
		m	73.27	78.48	83.70	88.92	94.14	97.81	101.50	105.17	108.85
		m%	1.00	1.07	1.14	1.21	1.28	1.33	1.39	1.44	1.49
		c	146.53	156.97	167.41	177.85	188.29	195.64	202.99	210.34	217.69
		c%	1.00	1.07	1.14	1.21	1.28	1.34	1.39	1.44	1.49
	1.2 N	a	146.55	156.96	167.40	177.84	188.29	195.62	202.99	210.34	217.70
		a%	1.00	1.07	1.14	1.21	1.28	1.33	1.39	1.44	1.49
		m	146.55	156.96	167.40	177.84	188.29	195.62	202.99	210.34	217.70
		m%	1.00	1.07	1.14	1.21	1.28	1.33	1.39	1.44	1.49
		c	293.06	313.94	334.82	355.70	376.57	391.27	405.98	420.68	435.37
		c%	1.00	1.07	1.14	1.21	1.28	1.34	1.39	1.44	1.49
	2.4 N	a	293.10	313.92	334.80	355.68	376.58	391.25	405.98	420.67	435.40
		a%	1.00	1.07	1.14	1.21	1.28	1.33	1.39	1.44	1.49
		m	293.10	313.92	334.80	355.68	376.58	391.25	405.98	420.67	435.40
		m%	1.00	1.07	1.14	1.21	1.28	1.33	1.39	1.44	1.49
		c	586.12	627.89	669.65	711.41	753.15	782.54	811.97	841.37	870.75
		c%	1.00	1.07	1.14	1.21	1.28	1.34	1.39	1.44	1.49
Translation	0.6 N	a	74.17	85.75	97.33	108.92	120.51	130.76	141.04	151.30	161.57
		a%	1.00	1.16	1.31	1.47	1.62	1.76	1.90	2.04	2.18
		m	74.17	85.75	97.33	108.92	120.51	130.76	141.04	151.30	161.57
		m%	1.00	1.16	1.31	1.47	1.62	1.76	1.90	2.04	2.18
		c	185.28	191.68	198.08	204.49	210.90	220.91	230.93	240.95	250.97
		c%	1.00	1.03	1.07	1.10	1.14	1.19	1.25	1.30	1.35
	1.2 N	a	148.33	171.50	194.66	217.84	241.01	261.53	282.07	302.60	323.14
		a%	1.00	1.16	1.31	1.47	1.62	1.76	1.90	2.04	2.18
		m	148.33	171.50	194.66	217.84	241.01	261.53	282.07	302.60	323.14
		m%	1.00	1.16	1.31	1.47	1.62	1.76	1.90	2.04	2.18
		c	370.57	383.35	396.17	408.98	421.80	441.82	461.87	481.90	501.94
		c%	1.00	1.03	1.07	1.10	1.14	1.19	1.25	1.30	1.35
	2.4 N	a	296.66	343.01	389.33	435.68	482.03	523.06	564.14	605.21	646.28
		a%	1.00	1.16	1.31	1.47	1.62	1.76	1.90	2.04	2.18
		m	296.66	343.01	389.33	435.68	482.03	523.06	564.14	605.21	646.28
		m%	1.00	1.16	1.31	1.47	1.62	1.76	1.90	2.04	2.18
		c	741.14	766.70	792.34	817.97	843.61	883.63	923.74	963.79	1003.88
		c%	1.00	1.03	1.07	1.10	1.14	1.19	1.25	1.30	1.35

a—apical third, m—middle third, c—cervical third (c—high amount of stress). a%, m%, c%—percentage stress increase.

## Data Availability

Data are contained within the article.

## References

[1] Shetty B., Fazal I., Khan S.F. (2022). FEA analysis of Normofunctional forces on periodontal elements in different angulations. Bioinformation.

[2] Moga R.A., Olteanu C.D., Buru S.M., Botez M.D., Delean A.G. (2023). Cortical and Trabecular Bone Stress Assessment during Periodontal Breakdown-A Comparative Finite Element Analysis of Multiple Failure Criteria. Medicina.

[3] Prados-Privado M., Martínez-Martínez C., Gehrke S.A., Prados-Frutos J.C. (2020). Influence of Bone Definition and Finite Element Parameters in Bone and Dental Implants Stress: A Literature Review. Biology.

[4] Hart N.H., Nimphius S., Rantalainen T., Ireland A., Siafarikas A., Newton R.U. (2017). Mechanical basis of bone strength: Influence of bone material, bone structure and muscle action. J. Musculoskelet. Neuronal Interact..

[5] Osterhoff G., Morgan E.F., Shefelbine S.J., Karim L., McNamara L.M., Augat P. (2016). Bone mechanical properties and changes with osteoporosis. Injury.

[6] Wang L., You X., Zhang L., Zhang C., Zou W. (2022). Mechanical regulation of bone remodeling. Bone Res..

[7] Tawara D., Nagura K. (2017). Predicting changes in mechanical properties of trabecular bone by adaptive remodeling. Comput. Methods Biomech. Biomed. Eng..

[8] Burr D.B. (2011). Why bones bend but don’t break. J. Musculoskelet. Neuronal Interact..

[9] Cicciù M., Cervino G., Milone D., Risitano G. (2018). FEM Investigation of the Stress Distribution over Mandibular Bone Due to Screwed Overdenture Positioned on Dental Implants. Materials.

[10] Moga R.A., Olteanu C.D., Botez M.D., Buru S.M. (2023). Assessment of the Orthodontic External Resorption in Periodontal Breakdown-A Finite Elements Analysis (Part I). Healthcare.

[11] Wu V., Schulten E., Helder M.N., Ten Bruggenkate C.M., Bravenboer N., Klein-Nulend J. (2023). Bone vitality and vascularization of mandibular and maxillary bone grafts in maxillary sinus floor elevation: A retrospective cohort study. Clin. Implant. Dent. Relat. Res..

[12] Moga R.A., Buru S.M., Olteanu C.D. (2022). Assessment of the Best FEA Failure Criteria (Part II): Investigation of the Biomechanical Behavior of Dental Pulp and Apical-Neuro-Vascular Bundle in Intact and Reduced Periodontium. Int. J. Environ. Res. Public. Health.

[13] Moga R.A., Buru S.M., Olteanu C.D. (2022). Assessment of the Best FEA Failure Criteria (Part I): Investigation of the Biomechanical Behavior of PDL in Intact and Reduced Periodontium. Int. J. Environ. Res. Public. Health.

[14] Moga R.A., Olteanu C.D., Daniel B.M., Buru S.M. (2023). Finite Elements Analysis of Tooth-A Comparative Analysis of Multiple Failure Criteria. Int. J. Environ. Res. Public Health.

[15] Hemanth M., Deoli S., Raghuveer H.P., Rani M.S., Hegde C., Vedavathi B. (2015). Stress Induced in the Periodontal Ligament under Orthodontic Loading (Part I): A Finite Element Method Study Using Linear Analysis. J. Int. Oral Health.

[16] Hemanth M., Deoli S., Raghuveer H.P., Rani M.S., Hegde C., Vedavathi B. (2015). Stress Induced in Periodontal Ligament under Orthodontic Loading (Part II): A Comparison of Linear Versus Non-Linear Fem Study. J. Int. Oral Health.

[17] Moga R.A., Olteanu C.D., Botez M., Buru S.M. (2023). Assessment of the Maximum Amount of Orthodontic Force for Dental Pulp and Apical Neuro-Vascular Bundle in Intact and Reduced Periodontium on Bicuspids (Part II). Int. J. Environ. Res. Public Health.

[18] Moga R.A., Olteanu C.D., Botez M., Buru S.M. (2023). Assessment of the Maximum Amount of Orthodontic Force for PDL in Intact and Reduced Periodontium (Part I). Int. J. Environ. Res. Public Health.

[19] Proffit W.R., Fields F.H., Sarver D.M., Ackerman J.L. (2012). Contemporary Orthodontics.

[20] Merdji A., Mootanah R., Bachir Bouiadjra B.A., Benaissa A., Aminallah L., el Ould Chikh B., Mukdadi S. (2013). Stress analysis in single molar tooth. Mater. Sci. Eng. C Mater. Biol. Appl..

[21] Field C., Ichim I., Swain M.V., Chan E., Darendeliler M.A., Li W., Li Q. (2009). Mechanical responses to orthodontic loading: A 3-dimensional finite element multi-tooth model. Am. J. Orthod. Dentofac. Orthop..

[22] Shaw A.M., Sameshima G.T., Vu H.V. (2004). Mechanical stress generated by orthodontic forces on apical root cementum: A finite element model. Orthod. Craniofacial. Res..

[23] Yamanishi Y., Yamaguchi S., Imazato S., Nakano T., Yatani H. (2014). Effects of the implant design on peri-implant bone stress and abutment micromovement: Three-dimensional finite element analysis of original computer-aided design models. J. Periodontol..

[24] Pérez-Pevida E., Brizuela-Velasco A., Chávarri-Prado D., Jiménez-Garrudo A., Sánchez-Lasheras F., Solaberrieta-Méndez E., Diéguez-Pereira M., Fernández-González F.J., Dehesa-Ibarra B., Monticelli F. (2016). Biomechanical Consequences of the Elastic Properties of Dental Implant Alloys on the Supporting Bone: Finite Element Analysis. BioMed Res. Int..

[25] Shash Y.H., El-Wakad M.T., Eldosoky M.A.A., Dohiem M.M. (2022). Evaluation of stress and strain on mandible caused using “All-on-Four” system from PEEK in hybrid prosthesis: Finite-element analysis. Odontology.

[26] Park J.M., Kim H.J., Park E.J., Kim M.R., Kim S.J. (2014). Three dimensional finite element analysis of the stress distribution around the mandibular posterior implant during non-working movement according to the amount of cantilever. J. Adv. Prosthodont..

[27] Aunmeungtong W., Khongkhunthian P., Rungsiyakull P. (2016). Stress and strain distribution in three different mini dental implant designs using in implant retained overdenture: A finite element analysis study. Oral Implantol..

[28] Merdji A., Bachir Bouiadjra B., Achour T., Serier B., Ould Chikh B., Feng Z.O. (2010). Stress analysis in dental prosthesis. Comput. Mater. Sci..

[29] Perez-Gonzalez A., Iserte-Vilar J.L., Gonzalez-Lluch C. (2011). Interpreting finite element results for brittle materials in endodontic restorations. Biomed. Eng. Online.

[30] Vikram N.R., Senthil Kumar K.S., Nagachandran K.S., Hashir Y.M. (2012). Apical stress distribution on maxillary central incisor during various orthodontic tooth movements by varying cemental and two different periodontal ligament thicknesses: A FEM study. Indian J. Dent. Res. Off. Publ. Indian. Soc. Dent. Res..

[31] McCormack S.W., Witzel U., Watson P.J., Fagan M.J., Groning F. (2017). Inclusion of periodontal ligament fibres in mandibular finite element models leads to an increase in alveolar bone strains. PLoS ONE.

[32] Reddy R.T., Vandana K.L. (2018). Effect of hyperfunctional occlusal loads on periodontium: A three-dimensional finite element analysis. J. Indian Soc. Periodontol..

[33] Jeon P.D., Turley P.K., Moon H.B., Ting K. (1999). Analysis of stress in the periodontium of the maxillary first molar with a three-dimensional finite element model. Am. J. Orthod. Dentofac. Orthop..

[34] Jeon P.D., Turley P.K., Ting K. (2001). Three-dimensional finite element analysis of stress in the periodontal ligament of the maxillary first molar with simulated bone loss. Am. J. Orthod. Dentofac. Orthop..

[35] Hohmann A., Wolfram U., Geiger M., Boryor A., Kober C., Sander C., Sander F.G. (2009). Correspondences of hydrostatic pressure in periodontal ligament with regions of root resorption: A clinical and a finite element study of the same human teeth. Comput. Methods Programs Biomed..

[36] Hohmann A., Wolfram U., Geiger M., Boryor A., Sander C., Faltin R., Faltin K., Sander F.G. (2007). Periodontal ligament hydrostatic pressure with areas of root resorption after application of a continuous torque moment. Angle Orthod..

[37] Wu J., Liu Y., Li B., Wang D., Dong X., Sun Q., Chen G. (2021). Numerical simulation of optimal range of rotational moment for the mandibular lateral incisor, canine and first premolar based on biomechanical responses of periodontal ligaments: A case study. Clin. Oral Investig..

[38] Wu J., Liu Y., Wang D., Zhang J., Dong X., Jiang X., Xu X. (2019). Investigation of effective intrusion and extrusion force for maxillary canine using finite element analysis. Comput. Methods Biomech. Biomed. Eng..

[39] Wu J.L., Liu Y.F., Peng W., Dong H.Y., Zhang J.X. (2018). A biomechanical case study on the optimal orthodontic force on the maxillary canine tooth based on finite element analysis. J. Zhejiang Univ. Sci. B.

